# Chilling-Induced Changes in Aroma Volatile Profiles in Tomato

**DOI:** 10.1007/s11947-015-1504-1

**Published:** 2015-03-25

**Authors:** Brian Farneti, Alberto Algarra Alarcón, Fotios G. Papasotiriou, D. Samudrala, Simona M. Cristescu, Guglielmo Costa, Frans J. M. Harren, Ernst J. Woltering

**Affiliations:** 1 Horticultural Supply Chains Group, Wageningen University, Droevendaalsesteeg 1, 6708 PD Wageningen, The Netherlands; 2 Life Science Trace Gas Facility, Department of Molecular and Laser Physics, Institute for Molecules and Materials, Radboud University, P.O. Box 9010, 6500 GL Nijmegen, The Netherlands; 3 Department of Agricultural Sciences, Bologna University, Via Fanin 46, 40127 Bologna, Italy; 4 Food & Biobased Research, Wageningen University and Research Centre, P.O. Box 17, 6700 AA Wageningen, The Netherlands

**Keywords:** *Solanum lycopersicum* L., PTR-MS, GC-MS, Volatile compounds, Chilling injury

## Abstract

Fruit and vegetables are regularly stored by consumers in the refrigerator at temperatures that may be well below the recommended storage temperatures. Apart from causing visible symptoms such as watery, sunken areas on the skin, chilling may also induce changes in fruit textural properties and flavour. The aim of this research was to investigate the effect of low temperature storage on tomato flavour and off-flavour production. To more closely mimic the real-consumer aroma perception while eating, in addition to the standard solid-phase microextraction gas chromatography–mass spectrometry (SPME/GC-MS) analysis, volatiles were also measured using a chewing device connected to a proton-transfer reaction–mass spectrometer (PTR-MS). Aroma volatiles were assessed in red ripe tomatoes of the cvs Cappricia RZ (round truss) and Amoroso RZ (cocktail truss) stored at refrigerator temperature (4 °C) and at higher temperatures (16 and 22 °C) for 20 days. The changes in aroma production were also monitored when the fruit was brought from room to refrigerator temperature and vice versa. After bringing the fruit from room to refrigerator temperature, the abundance of most volatiles was greatly reduced within 3 to 5 h, closely following the decrease in fruit temperature. When temperature was restored to room temperature following varying times of cold storage, the abundance of most volatiles increased again, but generally not to the original levels. Overall, the effects of low temperature storage on the decrease in volatile abundance were more pronounced in cv Cappricia RZ than in cv Amoroso RZ. On the contrary, the production of off flavours following prolonged cold storage was more pronounced in cv Amoroso RZ than in cv Cappricia RZ. Apart from changes in the overall abundance of the volatiles, marked changes in the volatile profile were observed in fruit stored for longer times in the cold and this may at least in part explain the negative effect of cold storage on overall tomato flavour.

## Introduction

Providing more tasty fruit and vegetables is likely to increase their consumption. More effort and attention should be paid to improve and optimize flavour upon delivery to the consumer, and flavour should be considered as a central trait to determine fruit postharvest quality. The end of flavour life, due to changes in sugar, acids and aroma volatiles and the development of off flavours (mainly caused by fermentative metabolism), often precedes the end of shelf life as determined by visual and textural features (Kader 2003, 2008). Fruit volatile compounds can be considered as the end products of fruit metabolic processes. Changes in fruit metabolism may cause unpredicted alteration of the fruit volatile composition during storage (Zhang et al. 2008). Metabolic pathways for volatile biosynthesis, including those derived from amino acids, fatty acids and carotenoids, are diverse and often highly integrated with other portions of both primary and secondary metabolism (Mathieu et al. 2009; Dudareva and Klempien 2013; Goff and Klee 2006). Postharvest abuses, such as harvesting too immature fruit, mechanical injury during sorting and packing, extreme atmospheres and improper temperature management have been related to altered aroma volatile profiles and altered flavour perception (McDonald et al. 1996; Sargent et al. 1997; Maul et al. 1998; Maul et al. 2000; Tasdelen and Bayindirli 1998; Fellman et al. 2003; Forney 2008; Boukobza and Taylor 2002; Mejia-Torres et al. 2009).

The influence of postharvest strategies for long-distance shipping and long-term storage on quality of red ripe tomato was intensively studied over the last years (Beckles 2012; Suslow and Cantwell 2009). Exposure to storage temperatures below 13 °C may induce significant chilling injury (CI) in tomato fruit. Severity of CI is dependent on the length of the exposure to cold temperature as well as on the ripening stage of the fruit (King and Ludford 1983; Saltveit 2002, 2005; Farneti et al. 2012a). One of the negative effects of chilling injury on quality is aroma degradation. As determined by quantitative descriptive analysis as well as GC-MS analytical quantification, tomatoes subjected to low-storage temperatures have lower levels of (ripe) aroma, lower levels of tomato flavour and higher levels of off-flavour compounds when compared to tomatoes undergoing non-chilling temperature treatments (Bai et al. 2011; Boukobza and Taylor 2002; Krumbein et al. 2004; Maul et al. 2000; Zhang, et al. 2008). Off-flavour compounds are thought to influence the perception of other aroma compounds: For instance, ethanol and methanol at higher concentrations have been found to suppress perception of certain tomato aroma volatiles, such as hexanal, 3-methylbutanol and phenylethanol, while enhancing perception of other volatiles, such as *trans*-2-hexenal, hexanol and 3-methylbutanal (Tandon et al. 2000; Abegaz et al. 2004).

Avoiding incorrect storage practices along all the distribution chain may not be sufficient to prevent fruit quality reduction since home refrigerator storage of a variety of chilling sensitive commodities such as tomato, cucumber and bell pepper is still common practice (Farneti et al. 2012a). Among evident quality deterioration, such as fruit discoloration, lycopene degradation, softening and skin pitting (King and Ludford 1983; Saltveit 2002, 2005; Farneti et al. 2012a), home refrigerator storage at around 4–6 °C may cause a severe alteration of the tomato aroma profile and it could be considered as one of the most contributing factors to consumer complaints about inferior tomato flavour (Maul et al. 2000). According to Bai et al. (2011), tomato placed at room temperature after a period of cold storage should recover aroma volatiles until some point without reaching the control level. However, those results were limited to six-carbon (C6) aldehydes, such as hexanal, Z-3-hexenal and E-2-hexenal and corresponding alcohols.

The aim of this research was to investigate the effects of low temperature storage on tomato aroma compounds with particular attention on the degree of aroma reversibility and the release of off-flavour volatiles after rewarming.

## Materials and Methods

### Fruit Sampling and Storage Treatments

Tomatoes of the cvs. Cappricia RZ (round truss) and Amoroso RZ (cocktail truss) from breeding company RijkZwaan BV, The Netherlands, were obtained in two production seasons (2010 and 2012) from a commercial greenhouse operation in the south-east of the Netherlands. Both cultivars were grown in the same greenhouse compartment under identical growing conditions. Homogenous batches of red ripe tomatoes from each cultivar were detached from the stem and selected on the base of fruit size and colour measured by a handheld photodiode array spectrophotometer (Pigment Analyzer PA1101, CP, Germany), according to Farneti et al. (2012a) and Schouten et al. (2014). The red ripe maturity stage was defined by Normalized Anthocyanin Index (NAI) values greater than 0.4 and 0.5 for the cvs Cappricia RZ and Amoroso RZ, respectively.

Tomato fruit of the first growing season (2010) were stored for 20 days at 4 ± 0.5 °C (RH of 93 ± 7 %) and at 16 ± 0.5 °C (RH of 88 ± 5 %) in the dark into commercial open carton boxes. Storage temperature was recorded hourly by a data logger (KeyTag KTL-108, Comtest, South Africa). At 5 days intervals (at 5, 10 and 15 days), a tomato batch prior stored at 4 °C was placed at 16 °C. Samples for volatile analysis by solid-phase microextraction gas chromatography–mass spectrometry (SPME/GC-MS) were taken at the starting time of the experiment (T0, within 24 h from harvest) every 5 days for the storage at 4 and 16 °C and after 1, 3 and 7 days after fruit were switched from 4 to 16 °C storage.

Tomatoes from the second growing season (2012) were stored for 12 days at 4 ± 0.5 °C (RH of 91 ± 5 %) and 22 ± 1 °C (RH of 94 ± 4 %) in the dark into commercial open carton boxes. Tomato samples stored at 4 °C and 22 °C were each day analysed for volatile production by using a chewing device coupled to a proton-transfer reaction–mass spectrometer (PTR-MS). The frequency of analysis was increased to five measurements a day, with a time interval of about 3 h, during the first 2 days after the fruit were brought from 22 to 4 °C and after the fruit were brought from 4 to 22 °C (after 6 and 12 days at 4 °C). Air storage temperature and tomato temperature were recorded by using thermo couples (Pico Tech., TC-08). For fruit temperature analysis, thermocouples were placed both on the skin and approximately 1 cm inside two tomatoes of each cultivar.

### SPME/GC-MS Analysis

Samples of fresh tomatoes (five fruits per cultivar for each temperature treatment) were quickly cut into quarters and immediately frozen in liquid nitrogen. The samples were stored at −80 °C and ground in liquid nitrogen in a metal electric grinder prior to analysis. The profiling of volatiles was performed using the headspace SPME/GC-MS method described by Tikunov et al. (2005), with slight modification (Farneti et al. 2012b). Frozen fruit powder (1 g fresh weight) was weighed in three replicates into a 20-mL crimp cap vial; the vial was closed and incubated at 30 °C for 10 min. The closed vials were then sonificated for 5 min. Thereafter, the samples were incubated at 60 °C with agitation for 30 min, and the headspace volatiles were extracted from the vial headspace and injected into the GC-MS apparatus (Trace GC Ultra, Thermo, IL, USA) equipped with a TriPlus SPME autosampler (Thermo, IL, USA). The extraction was done by inserting a Carboxen/PDMS SPME fibre (Supelco, Zwijndrecht, Netherlands) to the vial headspace during the last 20 min of the incubation under continuous agitation and heating at 60 °C. The fibre was desorbed for 15 min at 250 °C in the injection port of the GC in splitless mode. Chromatography was performed on RTX-WAX (Restek, Bellefonte, PA) capillary column (60 m length × 0.32 mm i.d., 0.25 μm film thickness) with helium as a carrier gas. The GC interface and MS (DSQ II, Thermo, IL, USA) source temperature were both 250 °C. The GC temperature program started at 40 °C (5 min) was then raised to 240 °C at a rate of 5 °C per min and, finally, was held at 240 °C for 10 min. The total run time including oven cooling was 60 min. Compound identification was based on mass spectra matching in the standard NIST library and retention time of authentic reference standards.

### PTR-MS Analysis

For tomato volatile analysis by PTR-MS, one intact tomato was placed into the 800-ml glass cuvette of the chewing device (Farneti et al. 2013) that was flushed with 1 L/h of clean air. Before crushing the fruit, the headspace volatile organic compounds (VOCs) concentration of the intact fruit was measured for 140 s to ensure reaching the equilibrium. The chewing was done through manually pressing the notched plunger five times within 10 s. VOC analysis continued for about 3 min following mastication. Each measurement was done in three replications. The head space was drawn from the chewing device at 0.5 L/h by a vacuum pump for online analysis into the PTR, as described by Farneti et al. (2012b). The PTR-MS apparatus consists of: (i) an ion source in which H_3_O^+^ ions are produced, (ii) a drift tube where the trace gases are ionized by proton-transfer reaction with H3O+ ions, (iii) a collision dissociation chamber, (iv) a quadruple mass filter and (v) a secondary electron multiplier. (for details, see Holger et al. 2012). The drift tube was operated at a pressure of 2.08 mbar and was heated at about 55 °C. The mass number of the detected ion is given by the molecular mass of the substance plus the mass of the single proton mH (mRH+ in atomic mass units).

Based on preliminary experiments with tomato fruits and based on earlier reported data (Farneti et al. 2012b), we selected and monitored 20 predominant masses from the overall tomato volatile spectrum (*m*/*z* 33, 41, 42, 43, 45, 47, 49, 51, 57, 59, 69, 81, 83, 84, 85, 87, 95, 99, 101 and 110). Mass spectrometric data were collected using a dwell time of 0.2 s per mass.

### Data Analysis

The multivariate statistical software Canoco 4.5 (Biometris-Plant Research International, Wageningen) was used for principal component analysis (PCA): Both GC-MS and PTR-MS data were LOG-transformed prior to the analysis.

## Result and Discussion

### VOCs Composition of Round and Cocktail Truss Tomato

In order to assess the effect of storage temperature on red ripe tomato volatile emission, tomatoes were first selected based on size and colour.

The volatile compound profile of round truss (cv Cappricia RZ) and cocktail truss tomato (cv Amoroso RZ) as analysed by SPME/GC-MS is shown in Table 1. The analysis allowed for the identification and quantification of 15 main compounds considered essential for the overall tomato aroma perception (Farneti et al. 2012b; Buttery et al. 1987, 1989; Krumbein and Auerswald 1998; Krumbein et al. 2004; Tandon et al. 2000). In accordance with Farneti et al. (2012b), red ripe tomatoes of cv Amoroso RZ were characterized by a 30 % higher volatile content (per FW unit) in comparison to the cv Cappricia RZ.

**Table 1:** Main volatile compounds of red ripe tomatoes of cvs. Amoroso RZ and Cappricia RZ assessed at harvest by SPME-GC-MS. Data are expressed in percentage of the total amount plus standard deviation (*n* = 3)

Volatile compound	Amoroso (%)	Cappricia (%)
Hexanal	58.05 ± 2.07	61.36 ± 3.24
*trans*-2-Hexenal	27.26 ± 0.94	19.19 ± 0.41
1-Nitro-pentane	5.08 ± 0.12	4.55 ± 0.18
3-Methylbutanal	3.24 ± 0.08	3.26 ± 0.05
5-Ethyl-2(5H)-furanone	1.73 ± 0.07	1.66 ± 0.05
Hexanoic acid	1.26 ± 0.02	1.63 ± 0.02
1-Penten-3-one	1 ± 0.01	1.92 ± 0.01
Pentanal	0.81 ± 0.02	1.43 ± 0.03
6-Methyl-5-hepten-2-one	0.64 ± 0.01	3.42 ± 0.05
*cis*-2-Heptenal	0.37 ± 0.01	0.46 ± 0.01
2-Cyclohexene-1,4-dione	0.29 ± 0.01	0.33 ± 0.01
Geranylacetone	0.1 ± 0.01	0.18 ± 0.01
*cis*-3-Hexenal	0.07 ± 0.00	0.1 ± 0.01
2-Isobutylthiazole	0.07 ± 0.00	0.47 ± 0.01
β-Ionone	0.03 ± 0.00	0.05 ± 0.00
Total concentration (μg/g)	314.8	209.6

It was confirmed that aldehydes are the most abundant volatiles in tomato, especially the ones derived from lipoxygenase activity: hexanal and *trans*-2-hexenal amount to more than 80 % of the total volatile headspace content of tomato. In addition, results confirmed that the ratio between hexanal and hexenals (*trans*-2-hexenal and *cis*-3-hexenal) varies in different tomato types with round truss tomato characterized by a higher value (3.2 for cv Cappricia RZ) than cocktail truss tomato (2.1 for cv Amoroso RZ). Despite their high levels and relatively low odour threshold concentrations, the importance of aldehydes for tomato liking has recently been questioned (Tieman et al. 2012; Farneti et al. 2013). Other compounds considered more important for the characteristic tomato flavour and liking are the compounds derived from carotenoids metabolism, such as geranyl acetone, β-ionone and 6-methyl-5-hepten-2-one (Goff and Klee 2006), and the ones derived from the amino acids leucine, isoleucine and phenylalanine such as methylbutanal (Klee and Tieman 2013; Tieman et al. 2006; Buttery and Ling 1993). Apart from the overall higher levels of aroma volatile in cv Amoroso RZ, the level of 6-methyl-5-hepten-2-one turned out to be a distinctive compound to discriminate the two tomato cultivars in this investigation. Based on absolute levels (in mg/g), the production of 6-methyl-5-hepten-2-one is about four times higher in Cappricia RZ compared to Amoroso RZ (Table 1). The relative headspace concentration of 6-methyl-5-hepten-2-one was much higher in cv Cappricia RZ (3.42 %) than in cv Amoroso RZ (0.64 %). For all the other compounds, no substantial differences (>2 times) in relative amount were observed (Table 1).

Volatiles measured using a SPME fibre do not necessarily fully reflect the actual head space composition of the tomato samples due to discrimination of volatiles on the basis of partition coefficients and adsorption kinetics (Berna et al. 2004). For instance, some relevant compounds such as methanol, ethanol and acetaldehyde that are highly present in tomato fruits were not detected (Farneti et al. 2012a, 2012b).

### VOC Changes During Storage Assessed by SPME/GC-MS

**Fig. 1:**
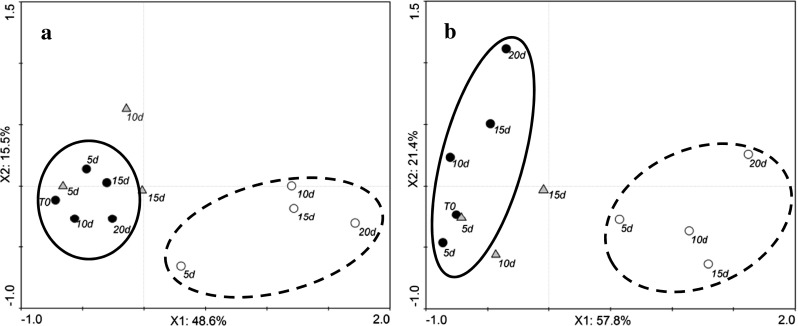
PCA scores of tomato volatiles assessed by SPME-GC-MS of the cvs Amoroso RZ (**a**) and Cappricia RZ (**b**). Measurements were done during storage for up to 20 days at 4 °C (*open circles*) and 16 °C (*filled circles*) and after 1 day of restoration at 16 °C following a storage period of 5, 10 and 15 days at 4 °C (*filled triangle*). The *number next to each point* indicates the number of days of storage. *Each point* is the average of three measurements of samples from a batch of five fruit. The data belonging to 16 and 4 °C have been *circled with a solid and dash line*, respectively

Tomato volatile profiles of both cvs Amoroso RZ and Cappricia RZ were significantly affected by the storage condition. Figure 1 shows the PCA biplots of the volatiles of tomatoes during storage measured by SPME/GC-MS. Variation of volatile content during storage is explained by the first two components for 64.1 and 79.2 % for cvs Amoroso RZ and Cappricia RZ, respectively. According to these results, red ripe fruit of cv Amoroso RZ exhibits a more stable volatile profile during the 20 days of storage at 16 °C in comparison to cv Cappricia RZ. This may be related to the sustained ripening during the storage period of cv Cappricia RZ tomatoes that were apparently not fully ripe at harvest, as indicated by an increase in red coloration during storage (data not shown). From the loading plots of the PCA analysis (data not shown), tomatoes of cv Cappricia RZ during the 16 °C storage were described by an increase in the concentrations of carotenoid-derived volatiles such as geranyl acetone and β-ionone whereas this was not observed in cv Amoroso RZ.

The VOC profile of 4 °C stored fruits significantly differed from 16 °C stored fruit. For both cultivars, the effect of 4 °C storage was similar: The longer the period of cold storage, the greater the distance between the 4 °C samples and the control in the PCA plot. These differences are mainly caused by a decrease in abundance of almost all the volatile compounds.

In accordance with Bai et al. (2011) and Boukobza et al. (2002), VOC profiling of fruit brought from 4 to 16 °C shows that the flavour recovery is negatively affected by the length of the period of cold storage: The longer the cold storage period, the less the recovery of the VOCs. This was more evident for cv Cappricia RZ than for cv Amoroso RZ. After 5 days at 4 °C, fruit of both cultivars shows an almost complete recovery of all volatiles whereas after 10 and 15 days at 4 °C, the recovery was only partial.

### VOCs Derived from Lipid Metabolism

**Fig. 2:**
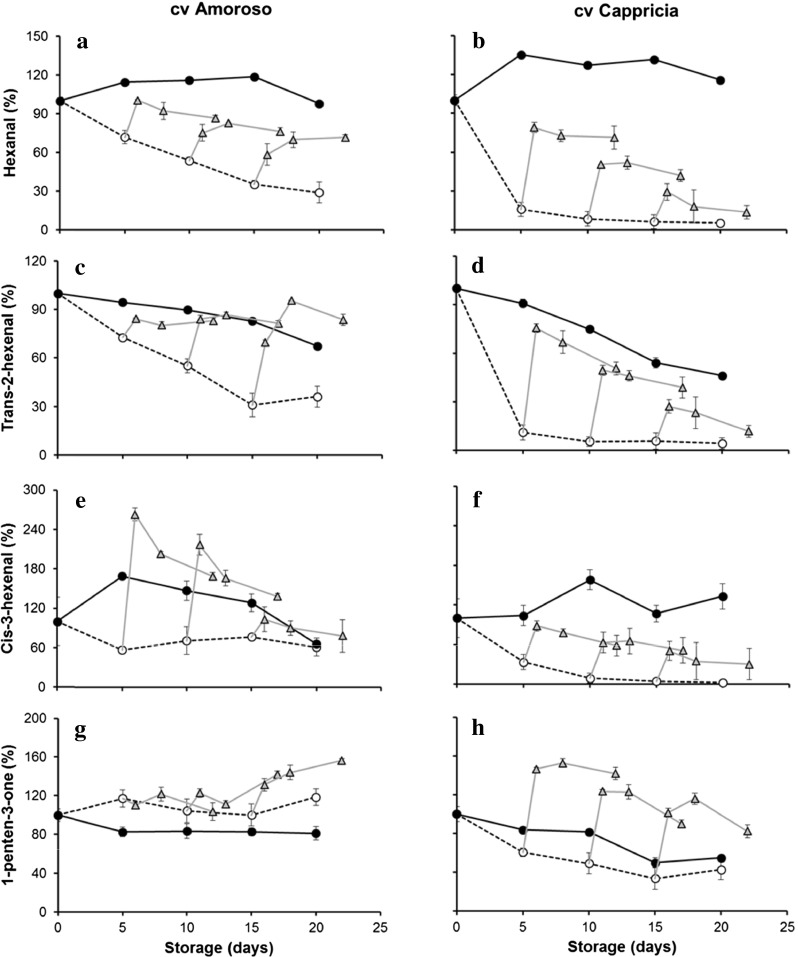
Volatiles derived from lipid metabolism assessed by SPME-GC-MS analysis of cvs Amoroso RZ (*left column* **a**, **c**, **e**, **g**) and Cappricia RZ (*right column* **b**, **d**, **f**, **h**). Measurements were done during storage for up to 20 days at 4 °C (*open circle*) and 16 °C (*filled circle*) and after restoration at 16 °C following a storage period of 5, 10 and 15 days at 4 °C (filled triangle). *Each point* is the average of three measurements plus standard deviation of samples from a batch of five fruit. SD bars are not visible; they were smaller than the symbol. Volatile abundance at *t* = 0 was set at 100 %

Dynamics of selected lipid-derived volatiles content during storage are shown in Fig. 2. Values, measured by SPME/GC-MS, are reported as a percentage relative to the initial concentration of the volatile at harvest (=100 %). The level of hexanal, being the most abundant volatile compound (about 60 % of total volatile concentration) in both cultivars, stored at 16 °C did not show an appreciable change.

Conversely, the level of *trans*-2-hexenal, the other main aldehyde of the tomato VOC profile, steadily decreased during the 16 °C storage period. After 20 days of storage, the concentration of *trans*-2-hexenal was only 70 and 50 % of the initial value in cvs Amoroso RZ and Cappricia RZ, respectively. On the contrary, the levels of the other lipid-derived VOCs, namely *cis*-3-hexenal and 1-penten-3-one, did not show appreciable change during storage at 16 °C. These results confirmed that tomato storage for long periods may reduce aldehyde content and, consequently, also the “freshness” flavour perception provided by these compounds (Bai et al. 2011; Krumbein et al. 2004; Maul et al. 2000).

Depletion of aldehydes was generally more severe during the storage at chilling temperature (4 °C), showing different patterns in fruit of the two tomato cultivars. A decrease in the two main aldehydes was observed; this decrease was more sudden and more drastic in cv Cappricia RZ than in cv Amoroso RZ. In the fruit of cv Amoroso RZ, the aldehyde content (hexanal and hexenals) decreased in a gradual and linear way during the 20 days of cold storage while in the fruit of the cv Cappricia RZ, the aldehyde content rapidly decreased within the first 5 days of storage. This effect of low temperature storage on volatile concentration was less evident for *cis*-3-hexenal and 1-penten-3-one.

Upon switching from 4 to 16 °C, production of volatiles increased and was stable within 1 day. Hexanal levels were generally not restored to original levels but, especially after a longer period at 4 °C, were much reduced. Levels of *trans*-2-hexenal and its isomeric form *cis*-3-hexenal were restored to levels similar to those of 16 °C stored fruit; levels of 1-penten-3-one, in both cultivars, showed an increase over the level in fruit at 16 °C.

According to Bai et al. (2011), the fruit of both cultivars resumed the production/emission of lipid-derived volatiles within 24 h when transferred from the storage at 4 to 16 °C, nevertheless without reaching the same level of the fruit stored continuously at 16 °C. The longer the cold storage duration at 4 °C, the lower the extent of aldehyde (particularly hexanal) formation when samples were transferred to 16 °C. Comparing the changes in aldehyde volatiles in response to temperature changes of cvs Amoroso RZ and Cappricia RZ tomatoes, cv Cappricia RZ appeared to be generally more sensitive to the low temperature storage. In cv Cappricia RZ, volatile production shows a more dramatic change after bringing the fruit to 4 °C as well as after bringing the fruit back to 16 °C. In cv Cappricia RZ fruit that has been stored for some time at 4 °C and thereafter brought to 16 °C, the levels of hexanal and hexenals are much lower and the level of 1-penten-3-one is much higher than in fruit continuously stored at 16 °C. From this observation, it may be expected that the fruit will have a less intense tomato flavour and the flavour may be less pleasant since 1-penten-3-one is commonly considered as a tomato off flavour due to the unpleasant organoleptic characteristics (Baldwin et al. 1998).

### VOCs Derived from Carotenoid Metabolism

**Fig. 3:**
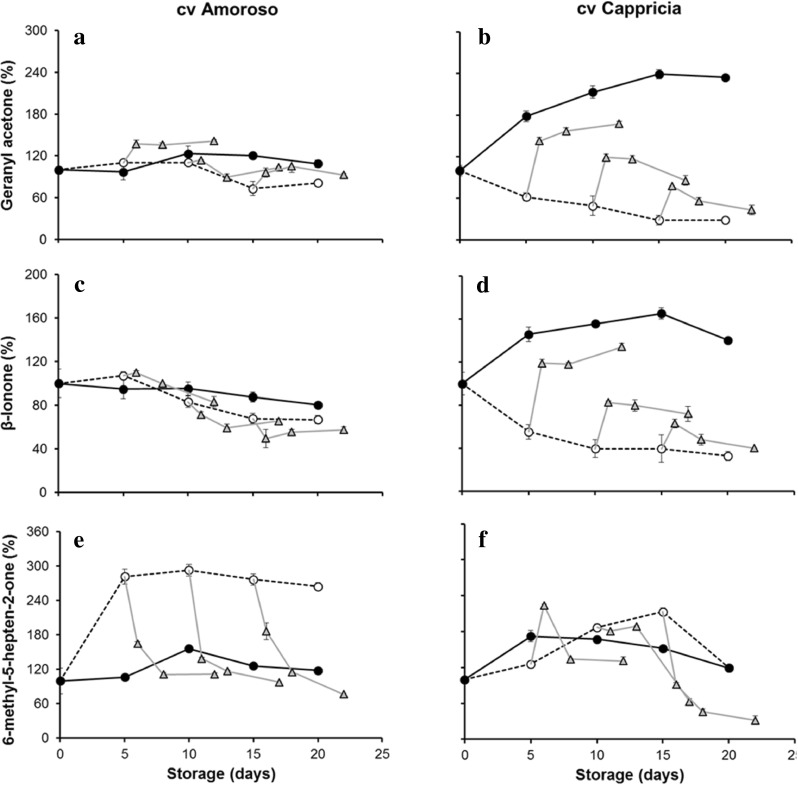
Volatiles derived by carotenoid metabolism assessed by SPME-GC-MS analysis of cvs Amoroso Z (*left column* **a**, **c**, **e**) and Cappricia RZ (*right column* **b**, **d**, **f**). Measurements were done during storage for 5, 10 and 15 days at 4 °C (*open circle*) and 16 °C (*filled circle*) and after restoration at 16 °C following a storage period of 5, 10 and 15 days at 4 °C (*filled triangle*). *Each point* is the average of three measurements plus standard deviation of samples from a batch of five fruit. SD bars are not visible; they were smaller than the symbol. Volatile abundance at *t* = 0 was set at 100 %

The effects of storage temperature on volatiles derived from carotenoid precursors, namely geranyl acetone, β-ionone and 6-methyl-5-hepten-2-one, are shown in Fig. 3. Compared to the lipid-derived volatiles, the response of carotenoid-derived aroma compounds to cold storage temperature was more cultivar dependent and less drastic. Geranylacetone and β-ionone content (Fig. 3a–d) were not noticeably affected by cold storage in the cv Amoroso RZ whereas fruit of cv Cappricia RZ showed already, after 5 days of storage at 4 °C, a reduction in the levels of these volatiles of over 50 %. Differences in volatile production between fruit stored at 4 and 16 °C increased throughout the 20 days of storage period in cv Cappricia RZ due to a continuous increase at 16 °C and a steady decline of the levels at 4 °C. The observed increase at 16 °C in the concentration of volatile compounds derived from the carotenoids lycopene and β-carotene may be a consequence of the sustained postharvest ripening of cv Cappricia RZ as reflected in the continued postharvest coloration of these fruit (data not shown). This indicates that, along with the production of lycopene and β-carotene, also the increased amounts of carotenoid-derived volatiles are produced. On the contrary, “Amoroso” fruit, evidently harvested at a more advances stage of ripening, did not show any significant increase in both red colour and carotenoid-derived volatiles. The production of 6-methyl-5-hepten-2-one (Fig. 3e, f), after bringing the fruit from 16 to 4 °C, showed a completely different pattern in the two cultivars. In cv Amoroso RZ, the production was about three times higher at 4 °C than at 16 °C while in cv Cappricia RZ, no clear effect of cold storage was observed.

When restored to 16 °C after a period of cold storage (4 °C), the production of geranyl acetone and β-ionone increased in the fruit of cv Cappricia RZ (Fig. 3b–d), but not to the levels of the fruit stored continuously at 16 °C. The longer the storage duration, the less restoration of volatile production was observed. In cv Amoroso RZ (Fig. 3a–c), no clear effect of the temperature change was seen.

A different trend was observed in 6-methyl-5-hepten-2-one following the temperature change from 4 to 16 °C (Fig. 3e, f). In cv Amoroso RZ fruit, the production decreased at 4 °C and was restored to the original levels within 2 days at 16 °C. In cv Cappricia RZ, the production also decreased at 4 °C but, as it had not increased after bringing the fruit to 4 °C; it now felt well below the original level.

**Fig. 4:**
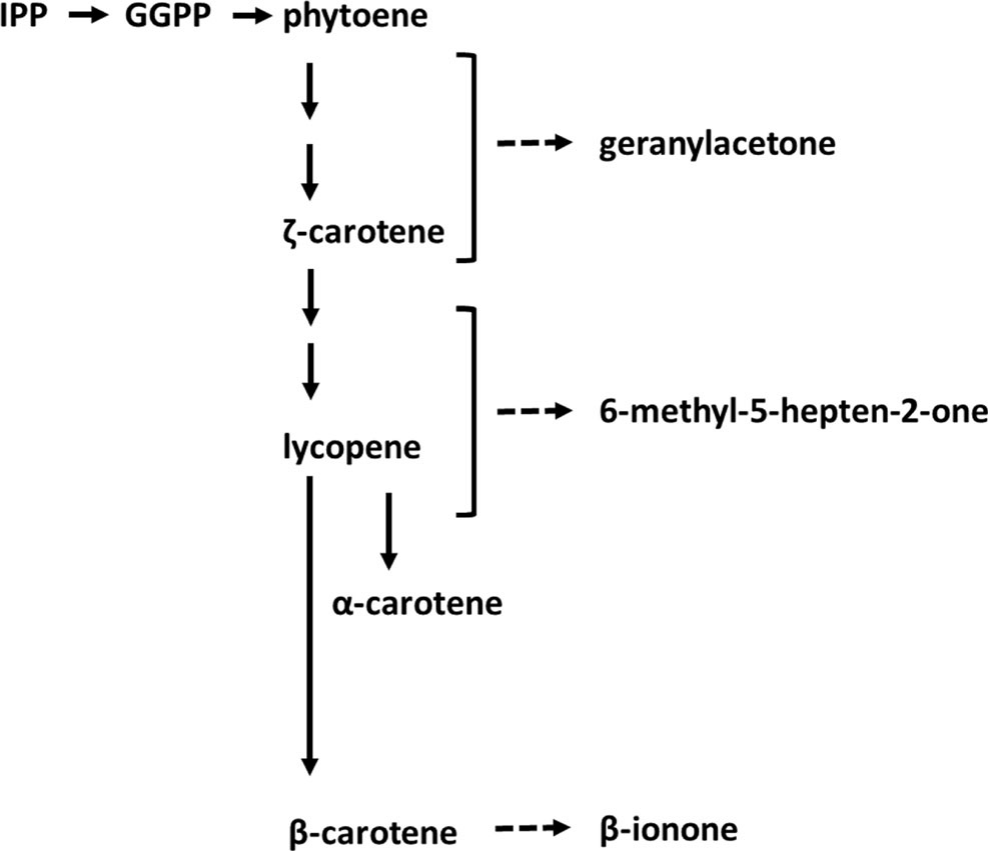
Scheme outlining the putative steps in carotenoid metabolism. Carotenoid biosynthesis begins with a C5-compound, isopentenyl-diphosphate (*IPP*) and its isomer dimethylally-diphosphate (DMAPP). Chain elongation leads to the formation of the C20-compound geranylgeranyl-diphosphate (*GGPP*). The head-to-head condensation of two GGPP molecules produces the first, colourless carotene, phytoene. A series of desaturation reactions lead to the coloured chromophore of lycopene, and subsequent cyclization reactions produce the β-carotene (adapted from Simkin et al., 2004 and Lewinsohn et al., 2005)

These results are consistent with the research of Farneti et al. (2012a) and Schouten et al. (2014) on the effect of low storage temperature (below 10 °C) on carotenoid synthesis and degradation in cocktail and round truss tomato (cv Amoroso RZ and Cappricia RZ). Following prolonged storage at chilling temperature, a decrease in lycopene content was observed due to a decreased synthesis and/or an increased breakdown (Farneti et al. 2012a; Schouten et al. 2014). Carotenoid-derived volatiles can be considered as degradation products of carotenoids or of carotenoid precursors. The reactions responsible for these conversions presumably take place in the chloroplasts and chromoplasts (in red fruit). At present, the exact biosynthetic pathways are not entirely known (Mathieu et al. 2009; Simkin et al. 2004). Geranylacetone is most likely derived from the compounds between phytoene and ζ-carotene (including ζ-carotene), 6-methyl-5-hepten-2-one from the compounds between ζ-carotene (excluding ζ-carotene) and α-carotene including lycopene and β-ionone is presumably derived from β-carotene (Fig. 4).

Particularly 6-methyl-5-hepten-2-one, production was shown to be correlated with phytoene synthase activity, a key enzyme in the synthesis pathway of lycopene. Indeed, this molecule can be synthetized from lycopene precursors such as prolycopene, δ-carotene and neurosporene (Mathieu et al. 2009; Tieman et al. 2006; Baldwin et al. 2000; Lewinsohn et al. 2005; Simkin et al. 2004) as well as from lycopene oxidation.

In the present experiments, at room temperature, the levels of the three apocarotenoid volatiles were stable over time in Amoroso RZ. As the Amoroso RZ fruit did not show any further colour development at room temperature, we may assume that the carotenoid synthesis and catabolism were in equilibrium and that the carotenoid biosynthesis pathway was operation at a steady-state level. On the contrary, Cappricia RZ tomatoes were harvested red but still showed further increase in lycopene during storage at room temperature. At room temperature, a significant increase in geranylacetone and β-ionone production was observed, indicating that, along with the sustained production of lycopene, also increased amounts of carotenoid-derived volatiles are produced.

During storage at low temperature, the production of 6-methyl-5-hepten-2-one considerably increased in Amoroso RZ fruit whereas the other carotenoid volatiles were not much affected (Fig. 3a–e). This indicates that in Amoroso RZ, the cold storage may specifically affect enzymes involved in conversion of lycopene or its precursors to 6-methyl-5-hepten-2-one. The gradual decrease in lycopene level during cold storage (Farneti et al. 2012a; Schouten et al. 2014) may be due to malfunctioning of one or more enzymes in the last steps of the pathway leading to lycopene. The increased synthesis of 6-methyl-5-hepten-2-one may in such a scenario be due to the accumulation of lycopene precursors and subsequent conversion into 6-methyl-5-hepten-2-one. When a tomato fruit is restored to room temperature, the last steps of lycopene synthesis may regain activity causing a decrease of 6-methyl-5-hepten-2-one in favour of lycopene building up. As the β-ionone concentration was not much affected by the low temperature storage in Amoroso RZ, this may indicate that the conversion of lycopene to β-carotene was not significantly affected by the low temperature. A diminished amount of lycopene or of its precursors and a continued breakdown of lycopene will lead to gradual loss of lycopene and of red colour. Following a switch from cold to room temperature, original levels of volatiles are restored, indicating that the effects of low temperature on carotenoid enzymes are reversible and that no permanent damage is done.

When Cappricia RZ is stored at low temperature, a decrease in geranylacetone and β-ionone was observed, but 6-methyl-5-hepten-2-one was not changed. This indicates a changed activity of enzymes in the carotenoid pathway. Considering the decrease in geranylacetone and β-ionone, the unchanged level of 6-methyl-5-hepten-2-one may be viewed as a relative “increase”. This may indicate that, as in Amoroso RZ, also in Cappricia RZ, the increased conversion of lycopene or its precursors to 6-methyl-5-hepten-2-one may at least partly explain the decreased lycopene levels in the cold. Following a switch from cold to room temperature, the levels of volatiles are not restored to original levels but stay lower than the original (also 6-methyl-5-hepten-2-one). This indicates that the carotenoid pathway in Cappricia RZ is more sensitive to cold than in Amoroso RZ as permanent loss of activity occurs. A similar difference in low temperature sensitivity between the two cvs was observed for the lipid-derived volatiles (Fig. 2).

### VOC Changes During Storage Assessed by PTR-MS and Chewing Device

While the aim of first experiment, based on SPME-GC-MS analysis, was to unravel the physiology and biochemistry behind the VOC formation at different storage temperatures, the second experiment aimed to simulate a situation more close to daily practice. For this reason, a different temperature regime was chosen, more close to the real-consumer home conditions (4 °C conservation and a rewarming phase at 22 °C) and a VOC assessment methodology that mimics the retronasal consumer perception during eating. Volatile profiling assessed by PTR-MS combined with a chewing device may give a more reliable reflection of real flavour perception during eating than the GC-MS measurements on frozen samples. The maceration procedure mimics the tissue breakdown that occurs in vivo during mastication while the PTR-MS is monitoring the development of flavour profile in a time span comparable to the food residence time in the mouth during chewing. Fruit volatile profile of cv. Amoroso RZ and Cappricia RZ, stored at 4 and 22 °C, were analysed by PTR-MS combined with a chewing device. The rapidity of this technique not only allowed more measurements along the storage period but also a more accurate monitoring of the VOC profile variations when storage temperature was changed from 4 to 22 °C.

**Fig. 5:**
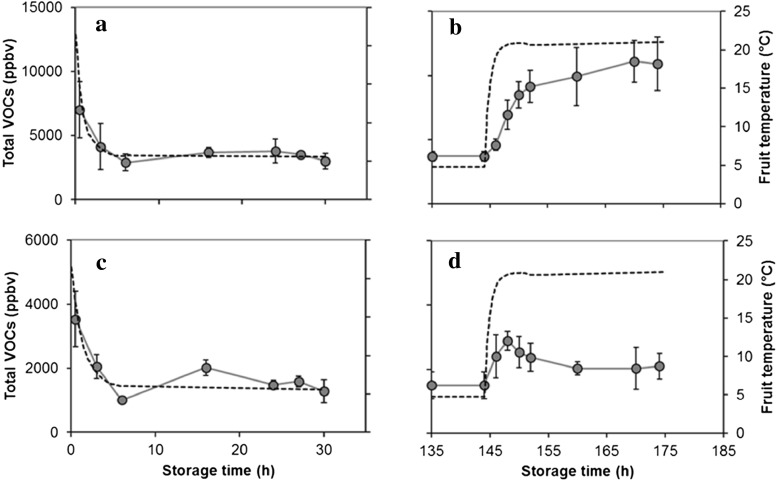
Comparison of internal fruit temperature (*black dashed line*) and the changes of total volatile production (*grey circles*) assessed by PTR-MS coupled with a chewing device of tomato fruits of cv Amoroso (**a**, **b**) and Cappricia (**c**, **d**). *Each point* is the average plus standard deviation of measurements of three tomatoes measured 30 s after the artificial mastication. SD bars are not visible; they were smaller than the symbol

Total volatiles emitted from the tomato matrix during mastication, as affected by fruit temperature, is shown in Fig. 5. The internal temperature of the fruit was influenced, as expected, by the volume/mass of the fruit with fruit of cv Amoroso RZ (around 30 gr of weight) characterized by a slightly faster change in temperature than cv Cappricia RZ (around 80 gr of weight). The decrease in the emission of volatiles followed with slight delay of the decrease in fruit temperature in both cvs (Fig. 5a–c). Within 5 to 6 h following the change from 22 to 4 °C, both the temperature and the volatile production were at a stable level. Following the change to 22 °C after 6 days at 4 °C, the increased production of volatiles followed, with some delay, the temperature change (Fig. 5b–d). The downregulation of volatile emission when fruit is brought from a high to a low temperature can be explained by an activity decline of the enzymes involved on volatile production (Bai et al. 2011) as well as a lower rate of volatilization. According to Henry’s law, the compounds will have a higher solubility at lower temperatures.

**Fig. 6:**
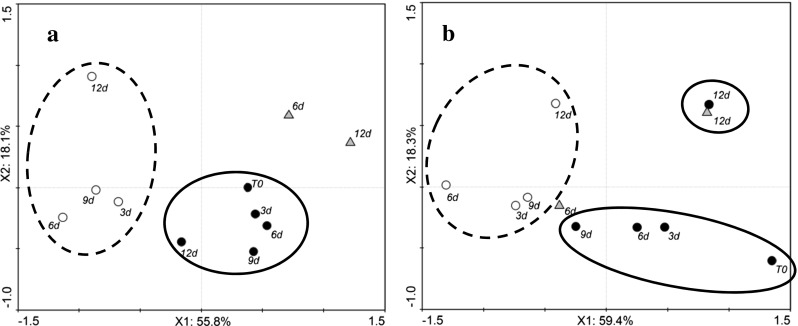
PCA scores of tomato volatiles assessed by PTR-MS (coupled with the chewing device) of the cvs. Amoroso RZ (**a**) and Cappricia RZ (**b**). Measured were done during 12 days of storage at 4 °C (*open circle*) and 22 °C (*filled circle*) and after 1 day of restoration at 22 °C following a storage period of 6 and 12 days at 4 °C (*filled triangle*). The *number next to each point* indicates the number of days of storage. *Each point* is the average of the measurements of three individual tomatoes measured 30 s after the artificial mastication. The data belonging to 22 and 4 °C have been *circled with a solid and dash line*, respectively

The volatile profiles of tomatoes of cvs Amoroso RZ and Cappricia RZ following storage for different times (3, 6, 9 and 12 days) at 22 and 4 °C and measured at 22 °C after 6 and 12 days of storage at 4 °C by PTR-MS coupled with the chewing device are shown in the PCA biplots (Fig. 6). Variations in volatile content during storage are explained by the first two components for 73.9 and 77.7 % for Amoroso RZ and Cappricia RZ, respectively. Similar to the result obtained by SPME/GC-MS analysis (Fig. 1), a separation of the tomato volatile profiles in two main clusters is noticeable, one for each storage temperature (4 and 22 °C). In addition, similarly to SPME/GC-MS results (Fig. 1), tomato of cv Amoroso RZ showed a more stable volatile profile during the 12 days of storage at 22 °C than cv Cappricia RZ. This may be a result of the more homogeneous and complete ripening stage of cv Amoroso RZ at harvest. The longer the storage time at 4 °C, the greater the distance to the control (t0) in the biplot for both cultivars.

Fruit kept at 4 °C for 6 and 12 days and then restored at 22 °C were characterized by a different volatile profile, not only determined only by overall lower compound concentration but also by an increased production of off flavours (Fig. 6).

**Fig. 7:**
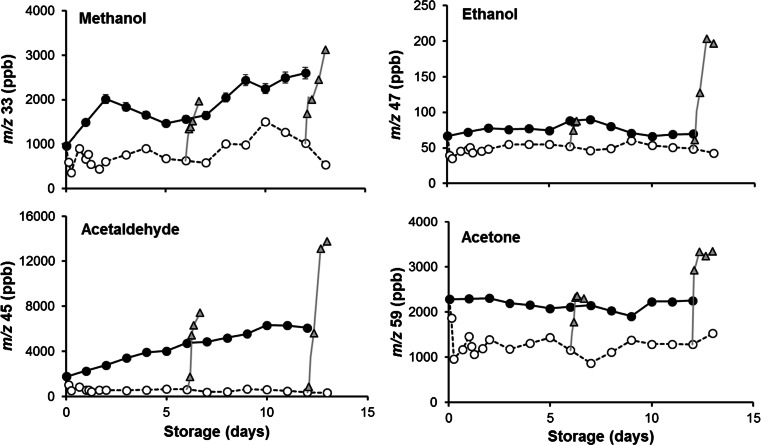
Off-flavour volatiles assessed by PTR-MS (coupled with the chewing device) of cv. Amoroso RZ. Measurements were done during 13 days of storage at 4 °C (*open circle*) and 22 °C (*filled circle*) and immediately after temperature restoration to 22 °C following a storage period of 6 and 12 days at 4 °C (*filled triangle*). *Each point* is the average of the measurements of three individual tomatoes plus standard deviation measured 30 s after the artificial mastication. SD bars are not visible; they were smaller than the symbol

**Fig. 8:**
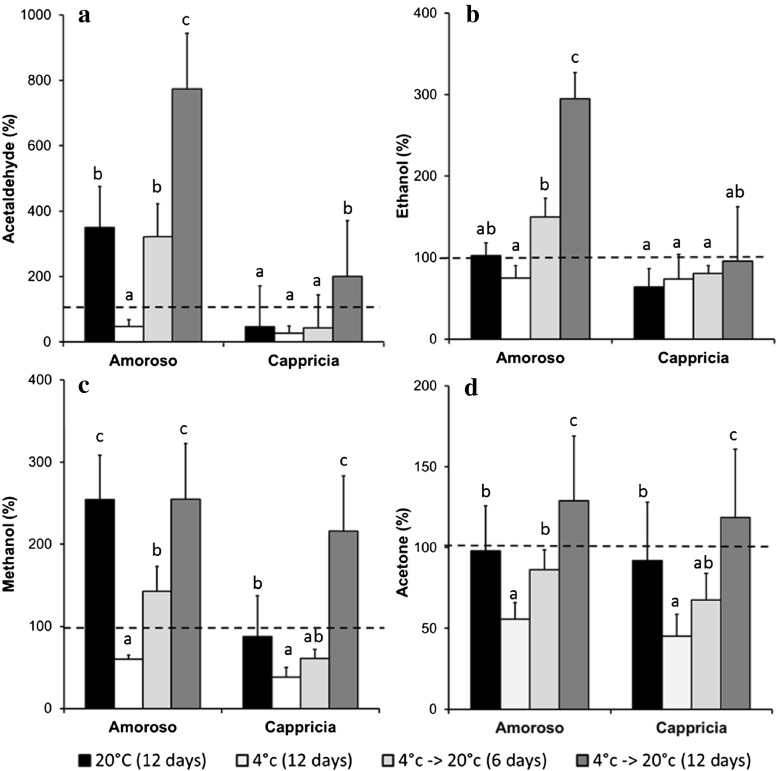
Off-flavour volatiles- acetaldehyde (**a**), ethanol (**b**), methanol (**c**), and acetone (**d**) - measured by PTR-MS (coupled with the chewing device) expressed as percentage of the initial value assessed at harvest (t0 = 100 %; dotted line). *Each point* is the average plus standard deviation of measurements of three individual tomatoes measured at 30 s after the artificial mastication. The prior low temperature-stored samples were assessed 1 day after restoration to room temp. *Different letters* within the same tomato cultivar indicate significant differences (LSD, *p* < 0.05)

Volatile patterns in fruit stored for 6 and 12 days at 4 °C and thereafter brought to 22 °C show a different behaviour in cv Amoroso RZ compared to cv Cappricia RZ (Fig. 6). According to the PCA biplots of the volatiles of the two cultivars, differences between these samples and the control were mainly explained by variation of the second principle component. According to loadings plots (not shown), the variation explained by the second principle component is mainly correlated with the concentration of compounds commonly associated with tomato off flavour, namely methanol, acetaldehyde, ethanol and acetone, masses 33, 45, 47 and 59, respectively. More detailed information on the concentration of these off-flavour compounds is presented in Figs. 7 and 8. The four off-flavour compounds had similar behaviour related to the storage temperature as the overall volatile patterns (Fig. 5): a decrease and increase along with the temperature changes. Concentration of VOCs is drastically reduced already after 6 h of cold storage, and thereafter, it stabilizes at a low level for the remaining cold storage period. Immediately after restoration to 22 °C, the production and release of all four off-flavour compounds restarted more evidently for the fruit of cv Amoroso RZ than cv Cappricia RZ (Figs. 7 and 8). The longer the storage period at 4 °C, the greater the off flavours production after the restoration at 22 °C. After 12 days of cold storage, production of acetaldehyde and ethanol in fruit of cv Amoroso RZ was almost doubled in comparison to the fruit stored constantly at 22 °C. Fruit of cv Cappricia RZ appeared more stable with less fluctuation with respect to off-flavour production related to temperature and storage period (Fig. 8).

Ethanolic fermentation is a two-step process in which pyruvate is first decarboxylated to acetaldehyde by pyruvate decarboxylase and acetaldehyde is subsequently converted to ethanol by alcohol dehydrogenase (Tadege et al. 1999). Acetaldehyde and ethanol are commonly considered as anaerobic fermentation products, but the ethanolic fermentation process can also take part in aerobic situation as a stress signal response, especially under abiotic stress such as dehydration or chilling (Tadege et al. 1999).

Methanol can be considered as well as an indirect stress response molecule since it can be produced from the hydrolysis of methyl ester groups in pectins: One of the most evident symptoms of senescence or induced stress in fruit, especially in tomato, is an increased rate of softening caused mostly by an increase of the pectin methyl esterase activity (Micheli 2001; Frenkel et al. 1998; Anthon and Barrett 2010). Tomatoes of the cv. Amoroso RZ were characterized by a higher rate of softening than cv Cappricia RZ (data not shown) at room temperature as well as at 4 °C that match with the higher methanol production (Fig. 7).

## Conclusion

A combination of sampling methods, including SPME-GC-MS and PTR-MS coupled with a chewing device, was used to study the volatile composition of cocktail and round truss tomato during cold storage and following restoration to room temperature. The aim of this research was to investigate the role of low storage temperature on tomato flavour and possible off-flavour production. Home refrigerator storage is still a common practice for the consumer to prolong the shelf life of fruit and, temperature abuse may also occur in the distribution chain. The emission of volatiles was related to the fruit temperature, with much lower total volatile emissions at 4 °C compared to room temperature suggesting that down- and upregulation of volatile emission by temperature can be explained either by a direct control of the enzymes activity involved on volatile production or by a control in the rate of volatilization.

Following restoration of temperature, volatile production generally went up again, but may reach lower levels than in fruit continuously stored at room temperature. In addition, the volatile profile of prior cold-stored fruit was different from fruits continuously stored at room temperature, with a relatively higher abundance of volatiles considered off flavours such as methanol, ethanol and acetaldehyde.
